# Urinary soluble urokinase receptor levels are elevated and pathogenic in patients with primary focal segmental glomerulosclerosis

**DOI:** 10.1186/1741-7015-12-81

**Published:** 2014-05-20

**Authors:** Jing Huang, Gang Liu, Yi-miao Zhang, Zhao Cui, Fang Wang, Xiao-jing Liu, Rong Chu, Ming-hui Zhao

**Affiliations:** 1 Renal Division, Peking University First Hospital, Beijing, PR China; 2 Institute of Nephrology, Peking University, Beijing, PR China; 3 Key Laboratory of Renal Disease, Ministry of Health of China, Beijing, PR China; 4 Key Laboratory of CKD Prevention and Treatment, Ministry of Education of China, Beijing, PR China; 5 Peking-Tsinghua Center for Life Sciences, Beijing, PR China

**Keywords:** Focal segmental glomerulosclerosis, Urinary soluble urokinase receptor, Podocyte

## Abstract

**Background:**

Focal segmental glomerulosclerosis (FSGS) is a major cause of end-stage renal disease. Recent studies have proposed that plasma soluble urokinase receptor (suPAR) might be a causative circulating factor but this proposal has caused controversy. This study aimed to measure urinary suPAR levels in patients with primary FSGS and its significance in the pathogenesis of FSGS.

**Methods:**

Sixty-two patients with primary FSGS, diagnosed between January 2006 and January 2012, with complete clinical and pathologic data were enrolled, together with disease and normal controls. Urinary suPAR levels were measured using commercial ELISA kits and were corrected by urinary creatinine (Cr). The associations between urinary suPAR levels and clinical data at presentation and during follow up were analyzed. Conditionally immortalized human podocytes were used to study the effect of urinary suPAR on activating β3 integrin detected by AP5 staining.

**Results:**

The urinary suPAR level of patients with primary FSGS (500.56, IQR 262.78 to 1,059.44 pg/μmol Cr) was significantly higher than that of patients with minimal change disease (307.86, IQR 216.54 to 480.18 pg/μmol Cr, *P* = 0.033), membranous nephropathy (250.23, IQR 170.37 to 357.59 pg/μmol Cr, *P* <0.001), secondary FSGS (220.45, IQR 149.38 to 335.54 pg/μmol Cr, *P* <0.001) and normal subjects (183.59, IQR 103.92 to 228.78 pg/μmol Cr, *P* <0.001). The urinary suPAR level of patients with cellular variant was significantly higher than that of patients with tip variant. The urinary suPAR level in the patients with primary FSGS was positively correlated with 24-hour urine protein (r = 0.287, *P* = 0.024). During follow up, the urinary suPAR level of patients with complete remission decreased significantly (661.19, IQR 224.32 to 1,115.29 pg/μmol Cr versus 217.68, IQR 121.77 to 415.55 pg/μmol Cr, *P* = 0.017). The AP5 signal was strongly induced along the cell membrane when human differentiated podocytes were incubated with the urine of patients with FSGS at presentation, and the signal could be reduced by a blocking antibody specific to uPAR.

**Conclusions:**

Urinary suPAR was specifically elevated in patients with primary FSGS and was associated with disease severity. The elevated urinary suPAR could activate β3 integrin on human podocytes.

Please see related article http://www.biomedcentral.com/1741-7015/12/82.

## Background

Focal segmental glomerulosclerosis (FSGS), which was first described by Rich in an autopsy series [1], is defined as a clinico-pathological syndrome manifesting with proteinuria, usually of nephrotic range, and pathological lesions of focal and segmental glomerular sclerosis and diffuse foot-process effacement [2,3]. FSGS is divided into either primary form (without known cause) or secondary to other kidney injuries, such as genetic variations, increased intra-glomerular pressures, reflux disease, viruses, drug toxicity and so on [4]. For primary FSGS, the etiology and pathogenesis have not been well elucidated, but the damage and detachment of podocytes from the glomerular basement membrane are regarded as the key in the initiation and progression of FSGS [5,6]. Previous studies have reported that secondary FSGS caused by genetic mutation usually did not recur following kidney transplantation, but in some patients with primary FSGS, it may recur within hours after kidney transplantation [7]. Some patients with primary FSGS were successfully treated with plasmapheresis or immunoabsorption [8,9], and a circulating permeability factor has been proposed in those patients [10,11]. Recently, Wei *et al.* found that soluble urokinase receptor (suPAR) might be the most likely causative circulating factor for primary FSGS [12]. Our previous study also revealed that elevated plasma suPAR might be specific for some patients with primary FSGS [13]. Other studies, however, have indicated that plasma suPAR might not be a specific marker for primary or idiopathic FSGS [14] and that it is unlikely to be the leading cause of childhood primary FSGS [15]. A recent study suggested that the urinary suPAR level might be a better biomarker than the plasma suPAR level in predicting the recurrence of FSGS after transplantation [16]. Our current study measured the urinary suPAR level in a variety of primary glomerular diseases, including primary FSGS with various pathological variants; we also analyzed its clinical significance and further investigated the possible pathogenic role of urinary suPAR in patients with primary FSGS.

## Methods

### Patients

According to the definition of primary FSGS in the Columbia classification [3], 62 patients with primary FSGS with complete clinical and pathological data, diagnosed in Peking University First Hospital between January 2006 and January 2012, were enrolled in this study. FSGS secondary to other primary glomerular diseases, such as IgA nephropathy, lupus nephritis, pauci-immune glomerulonephritis, membranous nephropathy, were excluded. All patients were negative for anti-neutrophil cystoplasmic antibody. The pathological variants of the 62 patients with primary FSGS include 19 tip variant, 21 not otherwise specified (NOS) variant, 20 cellular variant, 1 perihilar variant, and 1 advanced FSGS. The clinical and pathological data were collected at the time of presentation. Twenty eight patients had the last follow-up data, and none of them required kidney transplant. We collected urine samples from 16 patients with therapeutic responses.

Patients with nephrotic syndrome, defined as urinary protein excretion greater than or equal to 3.5 g/24 hours with serum albumin less than 30 g/L, were treated with corticosteroid combined with immunosuppressive agents including cyclophosphamide and cyclosporine A. Oral prednisone began at 1 mg/kg/day for up to 12 to 16 weeks followed by subsequent tapering, oral cyclophosphamide at 1.5 to 2 mg/kg/day for three months or cyclosporine A at 2 to 3 mg/kg/day with a trough concentration around 100 to 150 μg/ml, for 6 to 12 months. All patients were treated with angiotensin-converting enzyme inhibitors and/or angiotensin receptor blockers. For evaluation of the therapeutic response of patients with nephrotic syndrome, complete remission was defined as proteinuria less than or equal to 0.15 g/24 hours with stable serum Cr (no more than 25% increase in serum level from baseline). Partial remission was defined as proteinuria less than 3.5 g/24 hours but greater than 0.15 g/24 hours, with stable renal function in patients presenting with nephrotic syndrome. Treatment failure was defined as not reaching the criteria of partial remission. Patients who achieved partial remission and patients with treatment failure were collectively named the non-complete remission group.

Thirteen patients with minimal change disease, 22 patients with membranous nephropathy, 13 patients with secondary FSGS and 26 age- and gender-matched normal subjects were used as disease and normal controls. According to Hepinstall’s Pathology of the Kidney (6th Edition) [17], the pathologic diagnosis of secondary FSGS requires that a glomerular lesion falls within the morphologic spectrum of FSGS by light microscopy, but has segmental or a less severe degree of foot process effacement and/or electron dense deposits by electron microscopy, with or without clinically identifiable causes of FSGS. The 13 patients with secondary FSGS include 2 tip variant, 10 NOS variant and 1 perihilar variant according to light microscopy. Of the thirteen patients with secondary FSGS, there was one with pre-eclampsia, one with Kimura disease, two with obesity, one with Alport syndrome, and eight without identifiable factors. Compared to patients with primary FSGS, patients with secondary FSGS had less urine protein (3.7, inter-quartile range (IQR) 1.9 to 5.8 versus 7.7, IQR 5.4 to 13.3, *P* <0.001) and serum albumin levels in the normal range.

Informed consent was obtained from each patient. The design of this work was approved by the local clinical research ethics committee of Peking University First Hospital and was in compliance with the Declaration of Helsinki.

### Renal histopathology

Renal biopsy was performed at the time of diagnosis. Renal specimens were evaluated with direct immunofluorescence, light and electron microscopy, and were forwarded to two pathologists. Both pathologists examined the biopsies separately, being blinded to each other as well as to the patients’ clinical data. Differences in diagnosis between the two pathologists were resolved by re-reviewing the biopsies and coming to a consensus.

For direct immunofluorescence, immunoglobulin G (IgG), IgM, IgA, C3c, C1q, fibrinogen and albumin were detected by fluorescein isothiocyanate (FITC)-conjugated antibodies (Dako, Copenhagen, Denmark) on frozen tissues. The fluorescence intensity was determined using a semi-quantitative scale of 0 to 4+. For light microscopy, paraffin sections were stained with hematoxylin and eosin, periodic acid-schiff, periodic acid-silver methenamine and Masson’s trichrome. For electron microscopy, in brief, the tissue was fixed in 2.5% glutaraldehyde and 1% osmium tetroxide, then dehydrated in graded acetone and embedded in Epon 812. Ultrathin sections were cut at a thickness of 80 nm and placed on nickel grids. Then, the ultrathin sections were stained with uranyl acetate and examined by a transmission electron microscope JEM-1230 (JEOL, Tokyo, Japan).

### Sample collection

Spot urine samples of patients were collected in the first urination of the day of renal biopsy. The urine samples from 26 age- and gender-matched healthy donors were collected as normal controls. The urine, collected immediately after centrifugation at 1,800 rpm for 15 minutes at 4°C, was stored in aliquots at -80°C until use. Repeated freeze/thaw cycles were avoided. Urine samples from the 16 patients with follow up were also collected and stored until use.

### Quantification of urinary suPAR

We detected the concentration of urinary suPAR using the Quantikine Human uPAR Immunoassay (R&D Systems, Minneapolis, MN, USA) following the manufacturer’s protocol. In brief, the principle of the assay is a five-step procedure: (1) 96-well polystyrene microplates were pre-coated with a mouse monoclonal antibody against uPAR; (2) urine samples were diluted to 1:8 and added to each well and incubated for two hours at room temperature; (3) after incubation and washing, horseradish peroxidase-conjugated polyclonal antibodies against uPAR were added and incubated for two hours at room temperature; (4) after washing, substrate solution was added to each well and incubated for 30 minutes at room temperature protected from light; and (5) a stop solution was added to each well and then the absorbance was recorded using an enzyme-linked immunosorbent assay (ELISA) reader at 450/570 nm. The suPAR level of each sample was calculated using Curve expert 1.3.

### Cell culture

Conditionally immortalized human podocytes were kindly provided by Prof. Jochen Reiser (Rush University, Chicago, IL, USA). To propagate podocytes, cells were cultivated on Thermo Fisher Nunc plates (Thermo Fisher Scientific Inc, Slangerup, Denmark) at 33°C in the presence of Roswell Park Memorial Institute (RPMI)-1640 medium (Life Technologies Corp, Grand Island, NY, USA), containing 10% fetal bovine serum (Life Technologies Corp) and 1% insulin-transferrin-selenium (Life Technologies Corp). Cultured podocytes were seeded on coverslips (Thermo Fisher Scientific Inc) and allowed to differentiate for 14 days at the growth-restrictive temperature of 37°C in the presence of RPMI-1640 medium containing 10% fetal bovine serum before any treatment [18].

To study the activation effect of urinary suPAR on podocytes, conventional podocyte medium was changed to RPMI-1640 medium for 10 hours. Then, RPMI-1640 medium was changed into RPMI-1640 medium containing 5% urine from a patient with primary FSGS (a 44-year old female patient with primary FSGS and NOS variant, urinary suPAR levels: 450.98 pg/μmol Cr) at presentation, which had been filtered with a 0.22μm filter. Recombinant human suPAR protein (R&D Systems) was used at 1 μg/ml as a positive control [12]. RPMI-1640 medium containing 5% normal urine, 5% urine from a patient with minimal change disease (a 20-year old woman with a urinary suPAR level of 236.21 pg/μmol Cr) at presentation, 5% urine from a patient with membranous nephropathy (a 47-year old woman with a urinary suPAR level 215.37 pg/μmol Cr) at presentation, or 5% bovine serum albumin filtered with a 0.22 μm filter and RPMI-1640 medium containing nothing were used as controls. To investigate whether the FSGS urine-induced podocyte activation was due to the activation of the uPAR-β3 integrin pathway, monoclonal uPAR antibodies (R&D Systems, 1 μg/ml) were pre-incubated with the urine for one hour at 37°C in a water bath before being added to the podocytes. Forty-eight hours after treatment, human podocytes were fixed with 4% paraformaldehyde before immunofluorescence labeling. Urines from the other two patients with primary FSGS (NOS variant and cellular variant, respectively) with a higher level of urinary suPAR at presentation were investigated under the same conditions.

### Immunofluorescence staining of the activity of β3 integrin in human podocytes

The activity of β3 integrin of human podocytes is measured using the activation epitope–recognizing antibody AP5 (GTI Diagnostics, San Diego, CA, USA). In brief, fixed podocytes were washed three times with 0.01 Mol/L phosphate buffered saline, pH 7.4, permeabilized with 0.1% Triton X-100 (Sigma, Munich, Germany) in phosphate-buffered saline. To block non-specific staining, sections were incubated with 3% bovine serum albumin in phosphate-buffered saline for 30 minutes at room temperature. The primary antibody AP5 (dilution 1:50 in phosphate-buffered saline) was added to each coverslip directly. Antibodies were incubated overnight at 4°C. After sufficient wash with phosphate-buffered saline (pH 7.4) (five minutes, three times), Cy3-labeled donkey anti-mouse IgG (The Jackson Laboratory, West Grove, PA, USA, and dilution 1:400) was added and incubated for one hour at room temperature protected from light. The coverslip was then washed with phosphate-buffered saline and counter stained with 4’,6-diamidino-2-phenylindole (DAPI, 3 mM, Life Technologies Corp) for five minutes. Finally, sections were stored briefly at 4°C before being examined using an immunofluorescence microscope (Nikon Eclipse 80i, Tokyo, Japan). Negative controls were performed by omitting or replacing the primary antibodies. All the photos were taken with the same magnification (×400) and exposure time (800 milliseconds).

### Statistical analysis

Statistical analysis was performed using statistical software SPSS 16.0 (SPSS Inc., Chicago, IL, USA). Comparison of quantitative parameters was assessed using the non-parametric test between two non-normally distributed variables or one normally with one non-normally distributed variable. Comparison of paired variables was assessed using the paired samples *t* test (for data that were normally distributed) or paired samples non-parametric test (for data that were not normally distributed). Spearman’s correlation test was used to measure the correlation between two non-normally distributed variables or one normally with one non-normally distributed variable. All statistical analyses were two-tailed and *P* <0.05 was considered as significant.

## Results

### Demographic and clinical characteristics of patients with primary FSGS

The median age of the 62 patients with primary FSGS was 28 years, ranging from 13 to 84 years old. Forty-two were men and 20 were women. Their demographic, clinical and pathological data are listed in Table 1. Of the 62 patients, 59 had nephrotic syndrome (95.2%), 15 had acute kidney injury (24.2%), and 46 had microscopic hematuria (74.2%). Their median urine protein was 7.7 (IQR 5.4 to 13.3) g/24 hours. The mean serum albumin was 20.3 ± 5.9 g/L. The median serum creatinine was 89.0 (IQR 64.8 to 135.4) μmol/L at presentation.

**Table 1:** The demographic and clinical parameters of patients with primary FSGS

**Parameters**	**Number = 62**
Age (years), (median, range)	28, 13 to 84
Gender (male/female)	42/20
Nephrotic syndrome, number (%)	59 (95.2%)
Acute kidney injury, number (%)	15 (24.2%)
Microscopic hematuria, number (%)	46 (74.2%)
24-hour urine protein (g/24 hour) (median, IQR)	7.7, 5.4 to 13.3
Albumin (g/L) (mean ± s.d.)	20.3 ± 5.9
Serum creatinine at presentation (μmol/l) (median, IQR)	89.0, 64.8 to 135.4
Percentage of sclerosis in glomeruli (%) (mean ± s.d.)	16.1 ± 14.3
Percentage of segmental sclerosis in glomeruli (%) (mean ± s.d.)	11.7 ± 11.6
Percentage of global-sclerosis in glomeruli (%) (mean ± s.d.)	1.8 ± 4.1
Crescents formation, number (%)	9 (14.5%)

FSGS, focal segmental glomerulosclerosis; IQR, inter-quartile range; s.d., standard deviation.

### The urinary suPAR levels in patients with primary FSGS and controls

**Figure 1:**
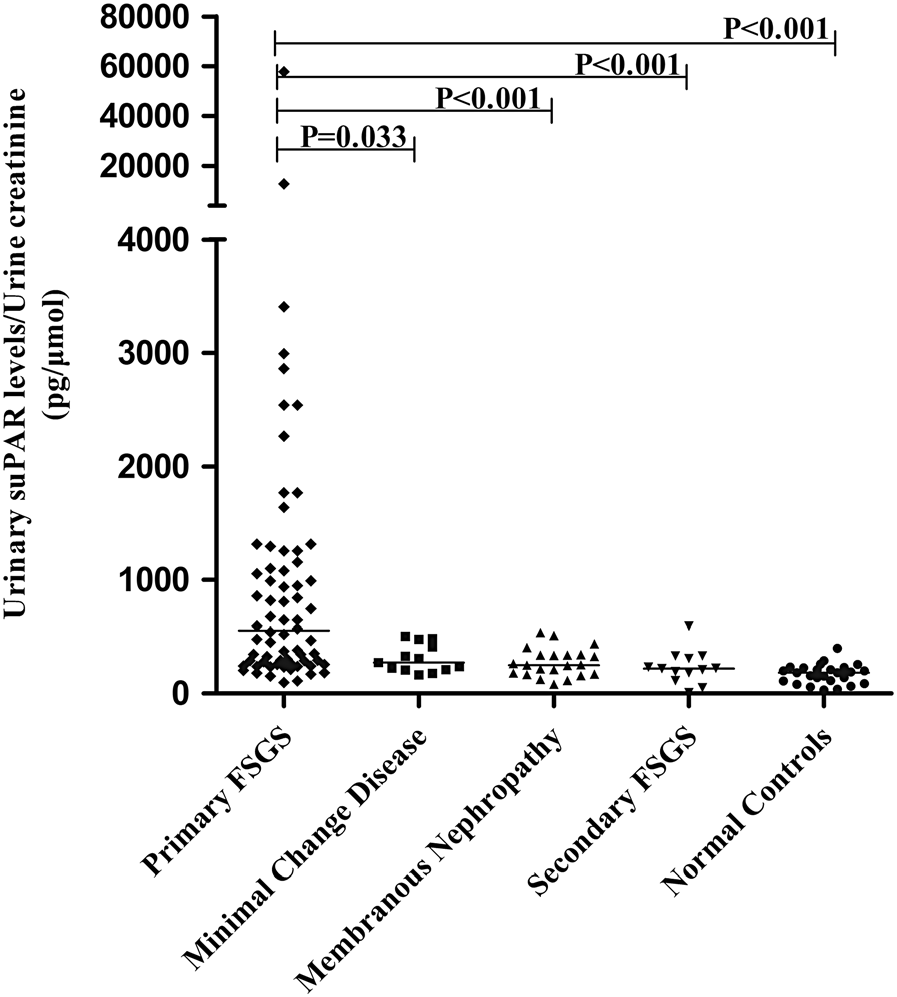
**Urinary suPAR levels among patients with primary FSGS, minimal change disease, membranous nephropathy, secondary FSGS and normal subjects.** FSGS, focal segmental glomerulosclerosis; suPAR, soluble urokinase receptor.

As molecular weights of suPAR fragments range from 22 to 45 kDa, these molecules could pass through the glomerular filtration barrier, and prior literature has also reported that the urinary suPAR level is correlated with the creatinine concentration [16]. So, in our study, the urinary suPAR levels were normalized by urinary creatinine levels to correct for differences in dilution, and the urinary suPAR levels were calculated by the ratio of urinary concentration of suPAR to the urinary creatinine concentration. The urinary suPAR levels of patients with primary FSGS, minimal change disease, membranous nephropathy, secondary FSGS and normal subjects are shown in Table 2 and Figure 1. The urinary suPAR level of patients with primary FSGS (500.56, IQR 262.78 to 1,059.44 pg/μmol creatinine (Cr)) is significantly higher than that of patients with minimal change disease (307.86, IQR 216.54 to 480.18 pg/μmol Cr, *P* = 0.033), membranous nephropathy (250.23, IQR 170.37 to 357.59 pg/μmol Cr, *P* <0.001), secondary FSGS (220.45, IQR 149.38 to 335.54 pg/μmol Cr, *P* <0.001) and normal subjects (183.59, IQR 103.92 to 228.78 pg/μmol Cr, *P* <0.001).

**Table 2:** The demographic data and urinary suPAR levels of patients and controls

	**Primary FSGS**	**Minimal change disease**	**Membranous nephropathy**	**Secondary FSGS**	**Normal control**
Number of subjects	62	13	22	13	26
Age (median, range)	28, 13 to 84	46, 18 to 71	50, 40 to 78	40, 15 to 46	27, 22 to 47
Gender (male/female)	42/20	6/7	15/7	4/9	10/16
Urinary suPAR/urine creatinine (pg/μmol) (median, IQR)	500.56, 262.78 to 1,059.44	307.86, 216.54 to 480.18	250.23, 170.37 to 357.59	220.45, 149.38 to 335.54	183.59, 103.92 to 228.78

FSGS, focal segmental glomerulosclerosis; IQR, inter-quartile range; suPAR, soluble urokinase receptor.

Using the mean plus two standard deviations of the urinary suPAR levels from the normal subjects as a cutoff value at 342.02 pg/μmol Cr, we found that 42 of the 62 patients with primary FSGS (67.7%) were above the cutoff value, but only 3 in the 22 with membranous nephropathy (13.6%), 4 in the 13 with minimal change disease (30.8%) and 1 in 26 normal controls (3.8%) were above the cutoff value.

### The association between plasma and urinary suPAR levels

Plasma suPAR levels of patients with primary FSGS and controls were measured and reported in our previous study [13]. There were 59 patients with primary FSGS, 13 patients with minimal change disease, 22 patients with membranous nephropathy, 13 patients with secondary FSGS and 26 normal subjects with both plasma and urinary suPAR levels. Plasma and urinary suPAR levels were positively correlated in patients with primary FSGS (r = 0.431, *P* = 0.001), but were not correlated in patients with minimal change disease (r = 0.121, *P* = 0.694), membranous nephropathy (r = 0.143, *P* = 0.526), secondary FSGS (r = 0.088, *P* = 0.775) nor normal subjects (r = 0.303, *P* = 0.132).

### The urinary suPAR levels in patients with primary FGGS with different pathological variants

**Figure 2:**
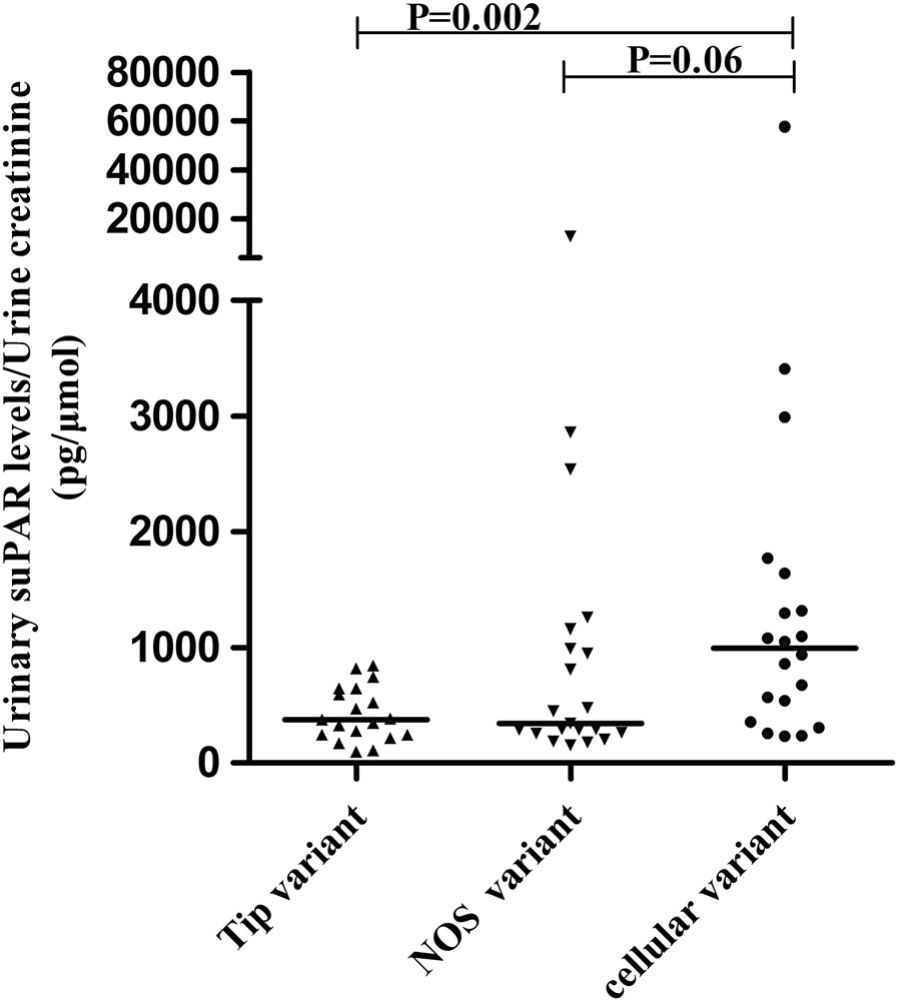
**The urinary suPAR levels in primary FSGS patients with different pathological variants.** FSGS, focal segmental glomerulosclerosis; suPAR, soluble urokinase receptor.

We compared the urinary suPAR levels among different histopathological variants of patients with primary FSGS. As shown in Figure 2, the urinary suPAR level of cellular variant (995.51, IQR 400.61 to 1,558.72 pg/μmol Cr) was significantly higher than that of tip variant (373.42, IQR 242.72 to 647.62 pg/μmol Cr, *P* = 0.002); although it was also higher than that of NOS variant, the difference was not significant (344.08, IQR 260.30 to 1,073.42 pg/μmol Cr, *P* = 0.06). The urinary suPAR level of the patient with perihilar variant was 2,269.13 pg/μmol Cr and the patient with advanced FSGS was 239.06 pg/μmol Cr.

Further analysis showed that nearly all the patients with tip variant had lower urinary suPAR levels which was comparable to that of minimal change disease (373.42, IQR 242.72 to 647.62 pg/μmol Cr versus 307.86, IQR 216.54 to 480.18 pg/μmol Cr, *P* = 0.367).

### The associations between urinary suPAR levels and clinical, laboratory data of patients with primary FSGS

**Figure 3:**
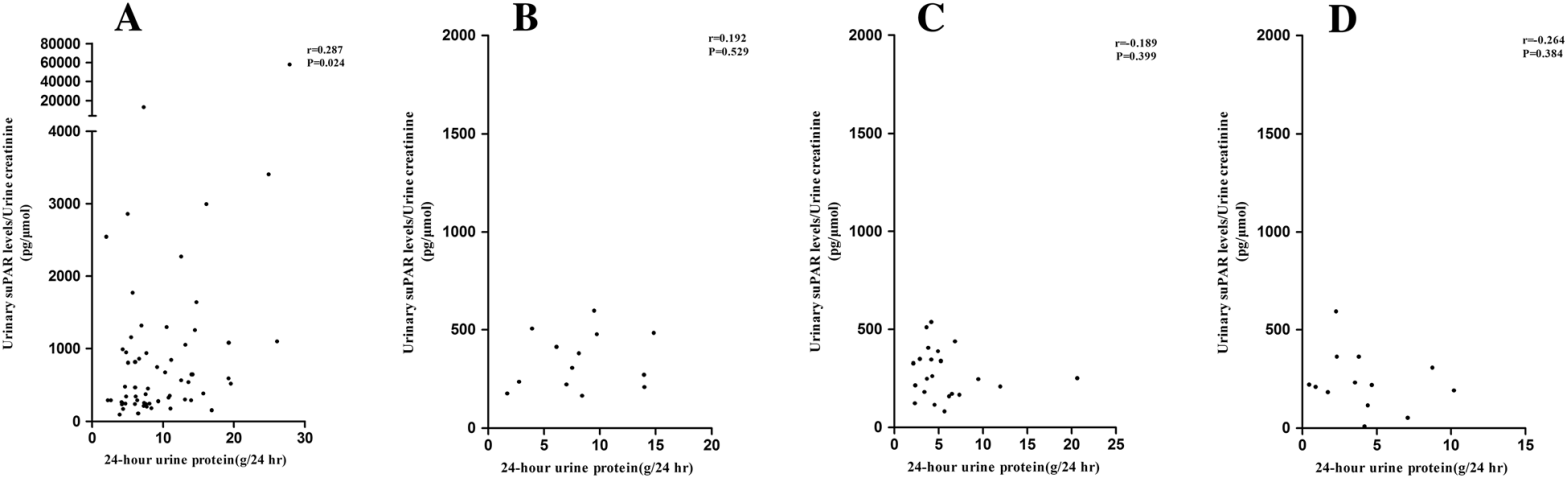
**The correlation between urinary suPAR levels and 24-hour urine protein. A**. Patients with primary FSGS; **B**. Patients with minimal change disease; **C**. Patients with membranous nephropathy; **D**. Patients with secondary FSGS. FSGS, focal segmental glomerulosclerosis; suPAR, soluble urokinase receptor.

In our study, it is of great interest to note that the urinary suPAR levels in patients with primary FSGS were positively correlated with 24-hour urine protein (r = 0.287, *P* = 0.024) (Figure 3A) and the erythrocyte sedimentation rate (r = 0.579, *P* <0.001) but were negatively correlated with plasma albumin (r = -0.269, *P* = 0.034) and hemoglobin (r = -0.400, *P* = 0.001). There was no significant correlation between urinary suPAR levels and C-reactive protein (CRP) (r = 0.242, *P* = 0.156). Furthermore, urinary suPAR levels in patients with minimal change disease (r = 0.192, *P* = 0.529) (Figure 3B), membranous nephropathy (r = -0.189, *P* = 0.399) (Figure 3C) and secondary FSGS (r = -0.264, *P* = 0.384) (Figure 3D) were not significantly correlated with 24-hour urine protein.

**Figure 4:**
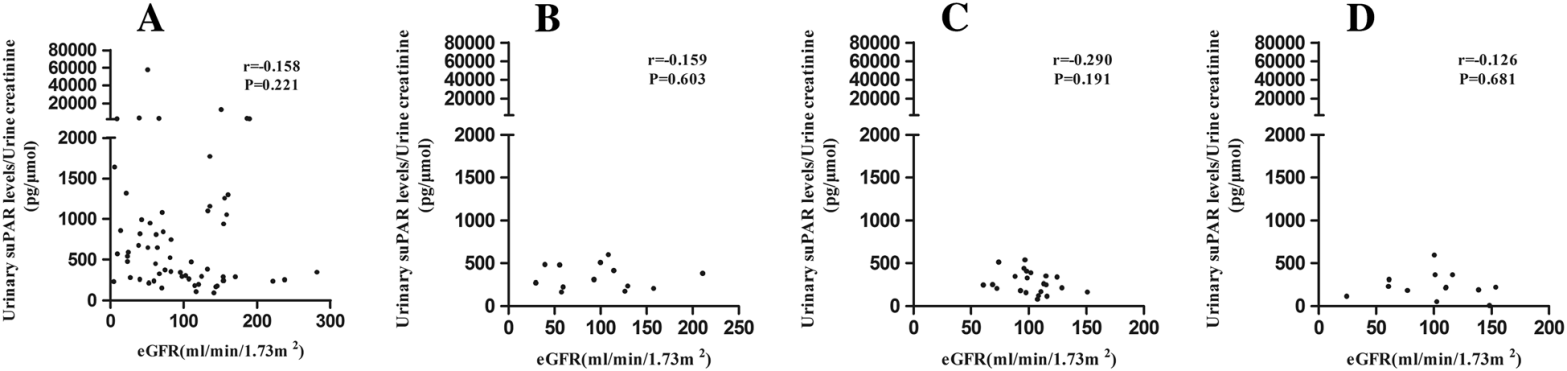
**The correlations between urinary suPAR levels and estimated glomerular filtration rate for each individual glomerular disease. A**. Patients with primary FSGS; **B**. Patients with minimal change disease; **C**. Patients with membranous nephropathy; **D**. Patients with secondary FSGS. FSGS, focal segmental glomerulosclerosis; suPAR, soluble urokinase receptor.

We further performed correlation analysis between urinary suPAR levels and estimated glomerular filtration rate (eGFR) for each individual glomerular disease (Figure 4). There was no correlation between the urinary suPAR levels and eGFR in patients with primary FSGS (r = -0.158, *P* = 0.221) (Figure 4A), minimal change disease (r = -0.159, *P* = 0.603) (Figure 4B), membranous nephropathy (r = -0.290, *P* = 0.191) (Figure 4C) or secondary FSGS (r = -0.126, *P* = 0.681) (Figure 4D).

### The change of urinary suPAR levels at presentation and during follow up of patients with primary FSGS

**Figure 5:**
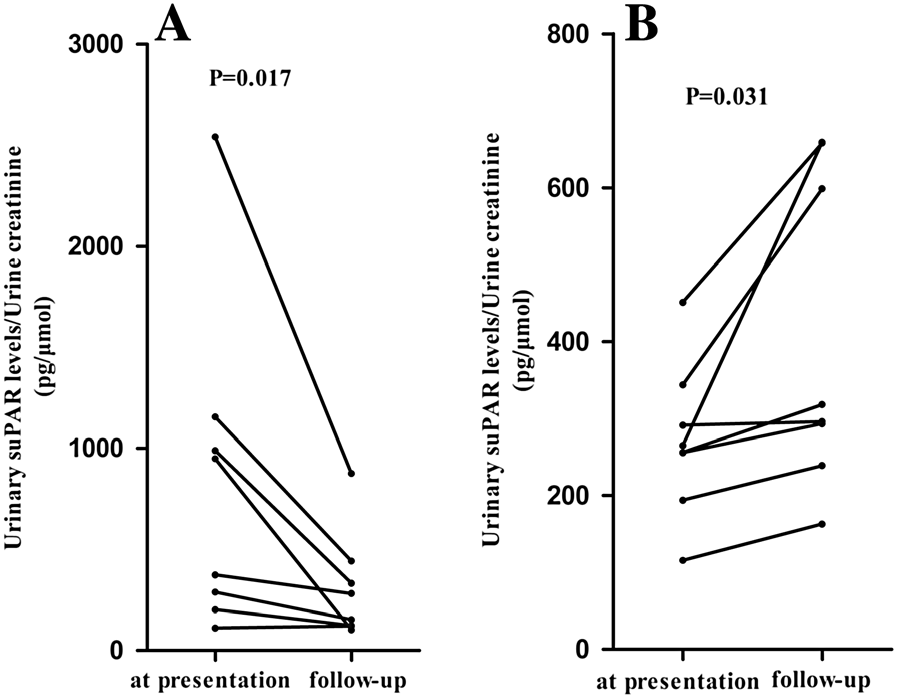
**The changes of urinary suPAR levels in patients with primary FSGS during follow up. A**. Patients with complete remission; **B**. Patients with non-complete remission. FSGS, focal segmental glomerulosclerosis; suPAR, soluble urokinase receptor.

During follow up with a mean duration of 80.0 (IQR 22.0 to 123.0) weeks, we collected urine samples from 16 patients with therapeutic responses. There were eight patients achieving complete remission, three patients achieving partial remission and five patients with treatment failure. At presentation, the median urinary suPAR levels of the three groups were not significantly different (661.19, IQR 224.32 to 1,115.29 pg/μmol Cr versus 265.00, IQR 115.73 to 291.77 pg/μmol Cr versus 256.14, IQR 224.88 to 397.53 pg/μmol Cr, *P* = 0.320, respectively). During follow up, the median urinary suPAR levels of the three groups were 217.68, IQR 121.77 to 415.55 pg/μmol Cr, 296.17, IQR 163.18 to 659.49 pg/μmol Cr and 318.22, IQR 266.32 to 628.65 pg/μmol Cr, respectively. In order to perform statistical analysis, we combined the partial remission group and the treatment failure group into a non-complete remission group. At presentation, the median urinary suPAR levels of patients achieving complete remission and non-complete remission were not significantly different (661.19, IQR 224.32 to 1,115.29 pg/μmol Cr versus 260.56, IQR 209.51 to 331.00 pg/μmol Cr, *P* = 0.093). Interestingly, the median urinary suPAR level of patients with complete remission decreased significantly (661.19, 224.32 to 1,115.29 pg/μmol Cr versus 217.68, IQR 121.77 to 415.55 pg/μmol Cr, *P* = 0.017) (Figure 5A), while for patients with non-complete remission, the mean urinary suPAR level increased significantly (271.68 ± 99.05 pg/μmol Cr versus 403.38 ± 201.55 pg/μmol Cr, *P* = 0.031) (Figure 5B). To compare with eGFR at presentation with that at follow up, we performed correlation analysis between the change of urinary suPAR levels and the change of eGFR in primary FSGS patients with complete remission (r = 0.214, *P* = 0.645) and non-complete remission (r = 0.571, *P* = 0.180). There was no correlation between the changes in suPAR and the changes in eGFR.

### Urinary suPAR could bind and activate β3 integrin in podocytes

**Figure 6:**
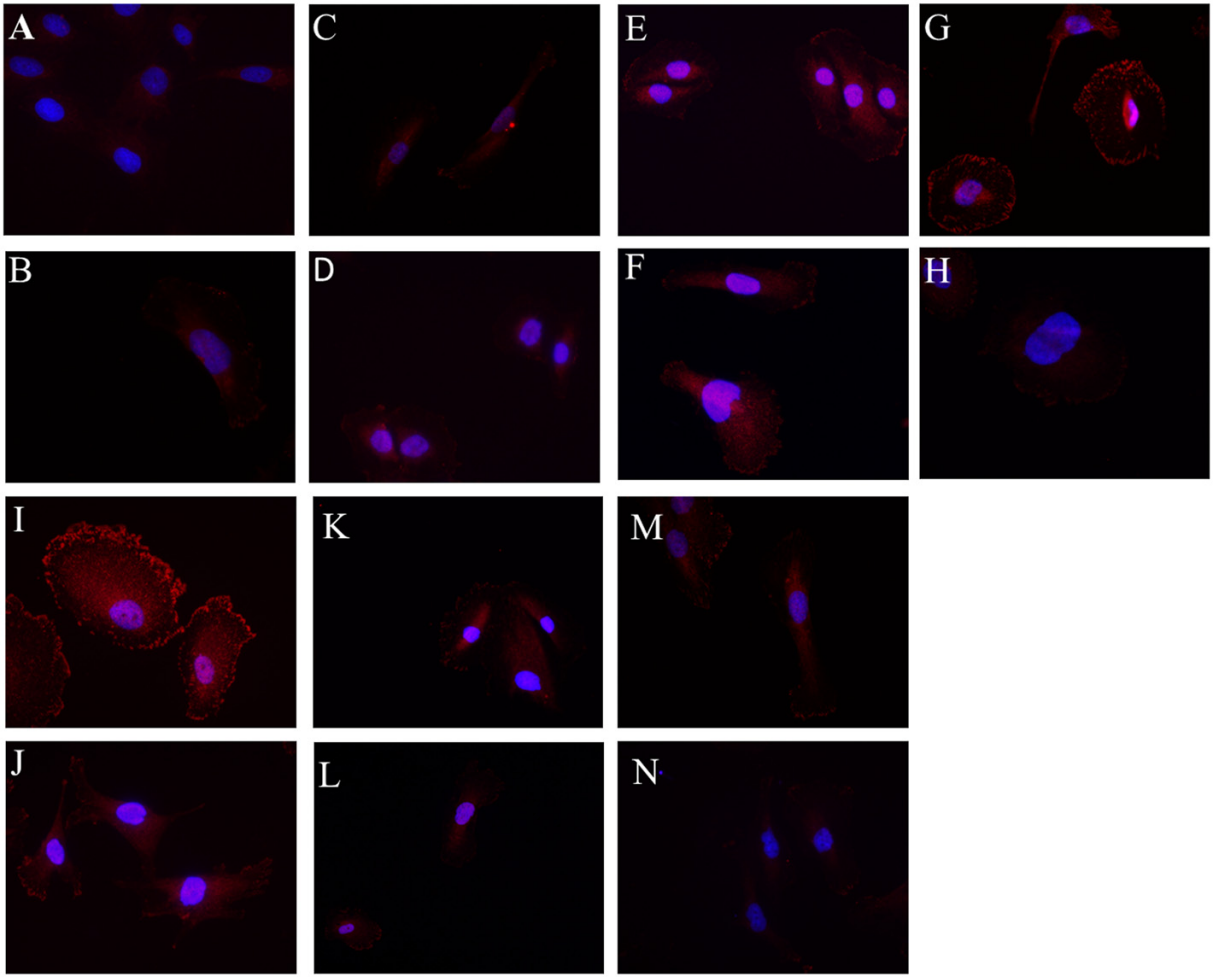
**AP5 immunofluorescence staining of differentiated human podocytes incubated with different additional materials. A**. AP5 antibody replaced by phosphate-buffered saline; **B**. Podocytes incubated with bovine serum albumin; **C**. Podocytes incubated with RPMI 1640; **D**. Podocytes incubated with RPMI 1640 combined with blocking antibody; **E**. Podocytes incubated with normal urine; **F**. Podocytes incubated with normal urine combined with blocking antibody; **G**. Podocytes incubated with recombinant human suPAR protein; **H**. Podocytes incubated with recombinant human suPAR protein combined with blocking antibody; **I**. Podocytes incubated with urine from patients with primary FSGS; **J**. Podocytes incubated with urine from patients with primary FSGS combined with blocking antibody; **K**. Podocytes incubated with urine from a patient with minimal change disease; **L**. Podocytes incubated with urine from a patient with minimal change disease combined with blocking antibody; **M**. Podocytes incubated with urine from a patient with membranous nephropathy; **N**. Podocytes incubated with urine from a patient with membranous nephropathy combined with blocking antibody. FSGS, focal segmental glomerulosclerosis; suPAR, soluble urokinase receptor.

In our study, we found that urinary suPAR levels in patients with primary FSGS were obviously higher than those of patients with minimal change disease, membranous nephropathy and normal subjects. Previous study has shown that suPAR can interact with β3 integrin. So we hypothesized that the elevated urinary suPAR levels in patients with primary FSGS might be pathogenic, and the urinary suPAR from patients with primary FSGS might induce podocyte injury via activating β3 integrin in cultured human podocytes.The human differentiated podocytes were incubated and cultured with urine from an index patient with primary FSGS at presentation. The recombinant suPAR, normal urine, bovine serum albumin and RMPI 1640 were used as controls. A blocking antibody to uPAR was used to confirm the activation effect of urinary suPAR on human podocytes. After 48 hour incubation, immunofluorescence staining was performed to analyze the expression and localization of the AP5 signal which corresponds to activated β3 integrin. The AP5 signal was strongly induced and expressed along the cell membrane when human differentiated podocytes were incubated with FSGS urine (Figure 6I) and recombinant suPAR (Figure 6G). However, the AP5 signal could be significantly reduced when FSGS urine and recombinant suPAR were previously incubated with the uPAR blocking antibody (Figure 6J, Figure 6H, respectively). Urines from the other two patients with primary FSGS with higher urinary suPAR levels at presentation confirmed the results. These data suggest that urinary suPAR could activate β3 integrin of human podocytes specifically. In contrast, the AP5 signal was very weak when human podocytes were incubated with normal urine (Figure 6E), urine from a patient with minimal change disease (Figure 6K), urine from a patient with membranous nephropathy (Figure 6M), bovine serum albumin (Figure 6B) and RPMI 1640 (Figure 6C), and the signal was not significantly influenced after the addition of the blocking antibody (Figure 6F, Figure 6L, Figure 6N, Figure 6D, respectively). Furthermore, the weak AP5 signal of podocytes incubated with RPMI 1640 combined with the blocking antibody (Figure 6D) indicated that this antibody did not influence the activation of β3 integrin. The details are shown in Figure 6.

## Discussion

FSGS is a major pathologic type of refractory nephrotic syndrome of children and adults, and a major cause of end-stage renal disease, with an estimated incidence of seven per million [2]. It is now regarded as a clinical–pathologic syndrome that has common glomerular lesions but is mediated by diverse causes. It includes a primary form of unknown causes and a form secondary to many other conditions, including drug toxicity, viruses, metabolic disorders and so on. During the past two decades, much evidence has revealed that there might be causative circulating factors in patients with primary FSGS [10,11]. In 2011, Wei *et al*. proposed serum suPAR as a possible cause of two-thirds of primary FSGS cases with renal transplantation for the first time [12]. They also validated the importance of serum suPAR in two discrete cohorts of children and young adults with biopsy-proven primary FSGS - the North America-based FSGS clinical trial (CT) and the Europe-based consortium for the study of steroid-resistant nephrotic syndrome (PodoNet) [19]. They also found increased circulating suPAR levels in a mother with FSGS and her newborn with a transient proteinuria [20]. Our previous study also revealed that plasma suPAR levels were elevated in more than half of patients with primary FSGS and were associated with treatment response [13]. However, the role of suPAR in primary FSGS has been a subject of controversy; some studies claimed that the serum suPAR level could not reliably predict response to treatment [14], and it was unlikely the leading cause for childhood primary FSGS [15]. Recently, Franco Palacios *et al*. reported that urinary suPAR levels but not serum suPAR levels in renal transplant recipients with FSGS recurrence before transplantation were higher than those in recipients with IgA nephropathy, membranous nephropathy, diabetic nephropathy and autosomal dominant polycystic kidney disease [16]. They indicated that the urinary suPAR level might be useful in predicting the recurrence of FSGS after transplantation [16]. Therefore, it is important to further investigate the role of suPAR in primary FSGS.

In the current study, we measured the urinary suPAR levels, on the same day as renal biopsy, in a Chinese cohort of patients with biopsy-proven primary FSGS and controls, and further analyzed the association between urinary suPAR levels and clinical parameters. Furthermore, we confirmed that suPAR in urine of patients with primary FSGS could induce β3 integrin activation in cultured human podocytes which leads to injury of the podocytes.

In the present study of our 62 patients with primary FSGS, their urinary suPAR levels were significantly higher than those of normal subjects, patients with minimal change disease, membranous nephropathy and secondary FSGS. In the same cohort of patients with primary FSGS, we found that the urinary suPAR levels of 67.7% patients with primary FSGS were above the cutoff value of normal subjects, but the plasma suPAR levels of only 54.1% were above the cutoff value. Further analysis indicated that the plasma suPAR levels and urinary suPAR levels were positively correlated in patients with primary FSGS, but not in disease controls and normal subjects. These data suggest that, in comparison with plasma suPAR, urinary suPAR might be a better biomarker to distinguish different diseases. More importantly, we found that the urinary suPAR levels at presentation were positively correlated with 24-hour urine protein and negatively correlated with plasma albumin levels in patients with primary FSGS. These data provide strong evidence that urinary suPAR might not only be a biomarker but also a pathogenic contributor to the pathogenesis of primary FSGS.

We have some speculations about the better differentiation performance of suPAR in urine than serum: 1) Urinary suPAR was presented as the ratio of suPAR over urine creatinine. This might make eGFR less a confounder in differential diagnosis. 2) Wei *et al.* and Zhang *et al*. have reported that the podocyte itself could produce lipid raft-associated uPAR and the uPAR-β3-integrin signaling participated in podocyte injury and proteinuria production [21,22]. Urinary suPAR levels represent both circulating and podocyte generated suPAR, so it may separate FSGS much more clearly. 3) suPAR consists of intact molecules and various segments. The pathogenic fragment has not been fully elucidated and commercial ELISA kits were used to detect all fragments. suPAR is a highly glycosylated protein. The glomerular filtration barrier might have charge selectivity to suPAR. The pathogenic suPAR might be more easily filtered through the glomerular basement membrane, binding on podocytes and concentrated in urine. This might make urinary suPAR more clinically significant. 4) The effect of suPAR on podocytes exhibited somewhat dose-dependent characteristics. We speculate that there might be a threshold for suPAR to induce podocyte injury in primary FSGS. The threshold might reduce the overlapping results shown in serum suPAR and enable the urinary suPAR to separate FSGS clearly.

FSGS has several pathological phenotypes or variants which might indicate various pathophysiological mechanisms. Our previous study indicated that the plasma suPAR levels, at the time of renal biopsy, were higher in patients with cellular variant than in those with tip and NOS variants, although the difference was not significant [13]. In this study, we found that urinary suPAR levels in patients with cellular variants were indeed significantly higher than those of patients with tip variant. Interestingly, our further analysis showed that the urinary suPAR levels were comparable between primary FSGS patients with tip variant and patients with minimal change disease. Previous studies had demonstrated that patients with tip variant showed the highest rate of treatment remission and lowest rate of end-stage renal disease compared with patients with other variants, while patients with cellular variant had higher proteinuria than patients with tip variant and NOS variant [23-25]. So we speculated that suPAR might be associated with different pathological lesions in patients with primary FSGS, which needs further investigation.

In order to demonstrate the pathogenic role of urinary suPAR in patients with primary FSGS, we investigated the activation effect of urinary suPAR on its ligand (AP5 staining), β3 integrin, in cultured human differentiated podocytes [21,22]. Our data showed that the AP5 signal was strongly induced and expressed along the cell membrane when podocytes were incubated with urine of patients with primary FSGS and recombinant suPAR, but the AP5 signal was very weak when podocytes were incubated with normal urine, bovine serum albumin or RPMI 1640. These results indicated that some soluble factor, probably elevated suPAR in the urine of patients with primary FSGS, actived the β3 integrin. More importantly, the urine-induced β3 integrin activation of podocytes could be reduced by a blocking antibody specific to uPAR. This demonstrated the specificity of urinary suPAR-induced β3 integrin activation in cultured human podocytes. Previous studies have proven that activation of β3 integrin is important to induce increased podocyte motility and foot process effacement caused proteinuria and FSGS in a mouse model [21]. So we could speculate that, at least from our study, suPAR in some patients with primary FSGS might induce podocyte injury via the activation of β3 integrin in podocytes, and this phenomenon suggests that suPAR might be one of the permeability factors for at least some patients with primary FSGS.

In this study, we also found that the urinary suPAR level at presentation was not associated with therapeutic response, which was consistent with Maas’s study and our previous study about plasma suPAR in patients with primary FSGS [13,14]. However, our previous study indicated that, in a small group of patients with treatment and follow up data, the elevated plasma suPAR could significantly decrease in patients who achieved complete remission [13]. In this study, it was found that the elevated urinary suPAR levels also decreased significantly in patients with complete remission, but not in those without complete remission. These findings indicated again that suPAR might be involved in the pathogenesis of primary FSGS. However, our studies had a relatively small size of patients with follow up data; a further large cohort study is needed to validate this phenomena.

Previous studies reported that suPAR levels were increased during infection and inflammation [26-29]. We analyzed the association between urinary suPAR levels and CRP, a sensitive biomarker of inflammatory status, but no correlation between urinary suPAR levels and CRP was identified, which is in accordance with a previous study [19]. This indicated that inflammation was not the major cause of the elevated urinary suPAR levels of our patients with primary FSGS.

uPAR, the glycosylphosphatidylinositol (GPI)-anchored protein with three domains (DI, DII, and DIII), is expressed on several different cell types, including neutrophils, monocytes, macrophages, activated T-lymphocytes, endothelial cells and kidney podocytes [30-32]. It could be released to plasma as suPAR after being cleaved of the GPI anchor, and it is also susceptible to cleavage at the linker region between DI and DII, so both the whole receptor and various segments of it are found free in the serum and are all called suPAR [33,34]. In addition to regulation of proteolysis, suPAR initiates signaling transduction in cooperation with other transmembrane proteins, such as integrins, caveolin and G-protein-coupled receptors, which promotes cell proliferation, invasion, motility and survival [35-37]. However, the pathogenic domain or part of the suPAR molecule in FSGS is not fully elucidated; it might be a specific domain, or a special form of glycosylation or phosphorylation of this interesting molecule. Although our results suggest that suPAR might play an important role in the pathogenesis of primary FSGS, there was still an overlap of urinary suPAR levels between patients with primary FSGS and patients with secondary FSGS and other glomerular diseases. In addition, various forms of the suPAR molecule exist in both plasma and urines in physiological conditions. However, the commercial ELISA kits used could not distinguish among these forms. Further studies are needed to identify the pathogenic part of the complex molecule and specific assays to detect pathogenic suPAR are needed.

## Conclusions

In conclusion, urinary suPAR was specifically elevated in patients with primary FSGS and was associated with disease severity. The urinary suPAR could activate β3 integrin of human podocytes.

## Competing interests

The authors declare that they have no competing interests.

## Authors’ contributions

JH carried out conception and design of this study, the collection, analysis and interpretation of data and drafted the manuscript. GL carried out conception and design of this study, renal pathological analysis and revised the manuscript. ZC carried out the follow-up of outpatients and revised the manuscript. YMZ and FW carried out the follow-up of outpatients. XJL and RC carried data collection. MHZ revised the manuscript and agreed to be accountable for all aspects of the work. All authors have read and approved the final manuscript.

## Pre-publication history

The pre-publication history for this paper can be accessed here:

http://www.biomedcentral.com/1741-7015/12/81/prepub
